# Evolution of rhodopsin ion pumps in haloarchaea

**DOI:** 10.1186/1471-2148-7-79

**Published:** 2007-05-18

**Authors:** Adrian K Sharma, David A Walsh, Eric Bapteste, Francisco Rodriguez-Valera, W Ford Doolittle, R Thane Papke

**Affiliations:** 1 Department of Biochemistry and Molecular Biology, Dalhousie University, 5850 College St., Halifax, Nova Scotia, B3H 1X5, Canada; 2 Unidad de Microbiologia, Centro de Biologia Molecular y Celular, Universidad Miguel Hernandez, Campus de San Juan, 03550 San Juan, Alicante, Spain

## Abstract

**Background:**

The type 1 (microbial) rhodopsins are a diverse group of photochemically reactive proteins that display a broad yet patchy distribution among the three domains of life. Recent work indicates that this pattern is likely the result of lateral gene transfer (LGT) of rhodopsin genes between major lineages, and even across domain boundaries. Within the lineage in which the microbial rhodopsins were initially discovered, the haloarchaea, a similar patchy distribution is observed. In this initial study, we assess the roles of LGT and gene loss in the evolution of haloarchaeal rhodopsin ion pump genes, using phylogenetics and comparative genomics approaches.

**Results:**

Mapping presence/absence of rhodopsins onto the phylogeny of the RNA polymerase B' subunit (RpoB') of the haloarchaea supports previous notions that rhodopsins are patchily distributed. The phylogeny for the bacteriorhodopsin (BR) protein revealed two discrepancies in comparison to the RpoB' marker, while the halorhodopsin (HR) tree showed incongruence to both markers. Comparative analyses of bacteriorhodopsin-linked regions of five haloarchaeal genomes supported relationships observed in the BR tree, and also identified two open reading frames (ORFs) that were more frequently linked to the bacteriorhodopsin gene than those genes previously shown to be important to the function and expression of BR.

**Conclusion:**

The evidence presented here reveals a complex evolutionary history for the haloarchaeal rhodopsins, with both LGT and gene loss contributing to the patchy distribution of rhodopsins within this group. Similarities between the BR and RpoB' phylogenies provide supportive evidence for the presence of bacteriorhodopsin in the last common ancestor of haloarchaea. Furthermore, two loci that we have designated bacterio-opsin associated chaperone (*bac*) and bacterio-opsin associated protein (*bap*) are inferred to have important roles in BR biogenesis based on frequent linkage and co-transfer with bacteriorhodopsin genes.

## Background

The halophilic archaea (haloarchaea) are aerobic heterotrophs that dominate hypersaline ecosystems suRch as the Dead Sea, Great Salt Lake and solar saltern facilities. Such ecosystems are frequently found in arid climates characterized by low precipitation levels and intense exposure to sunlight, which can lead to salinity levels as high as ten times that of sea water [1]. Worldwide, hypersaline environments represent a broad range of niches, which is reflected in the physiological properties of cultivated haloarchaea. In addition to aerobic respiration, a number of haloarchaea can ferment arginine, while others can use sulfur, DMSO or nitrate as alternative electron acceptors. Perhaps haloarchaea are most famous for their ability to generate a proton gradient through the use of photo-reactive rhodopsin proteins that harness light energy [2-5]. The type 1 (microbial) rhodopsins are typically seven-pass transmembrane proteins that use a retinal chromophore to absorb light energy for ion transport or photosensory functions [6]. First discovered in the haloarchaea in the early 1970's [7], genomic and metagenomic sequencing later revealed homologs in many disparate eukaryotes and bacteria, and most recently in the marine group II euryarchaeotes [8].

Four functional types of haloarchaeal rhodopsins are known. The H^+ ^pump bacteriorhodopsin (BR) uses light energy to create a proton electrochemical gradient for ATP production, flagellar rotation and other energy requiring processes [7]. The light-driven transport of chloride ions into the cytoplasm by the Cl^- ^pump halorhodopsin (HR), works to increase the electrochemical potential of the proton gradient, while the two types of sensory rhodopsins present in the haloarchaea are used for phototaxis under alternative maximum wavelengths of light (λ_max_) [9-13].

The model haloarchaeaon for rhodopsin studies, *Halobacterium *sp. NRC-1 exhibits differential expression patterns of its rhodopsins based on oxygen levels [2]. When oxygen is abundant, the expression of sensory rhodopsin II (SRII) facilitates avoidance motility from sunlight presumably to protect itself from harmful photo-oxidative damage. Under micro-oxic conditions *Halobacterium *sp. NRC-1 expresses three of its four rhodopsin types to harness solar energy and support periods of phototrophic growth. Sensory rhodopsin I (SRI) directs phototaxis of the cell into those wavelengths of light where both BR and HR absorb maximally, and can be used most efficiently. *Halobacterium *sp. NRC-1 is also one of the few haloarchaea known to produce a specialized area of its cell surface, called the purple membrane, where BR is the only protein present and covers as much as 50% of the cell surface [14].

Not all BR-producing haloarchaea produce purple membranes, however, and BR genes are themselves patchily-distributed among haloarchaea, as are genes for the different rhodopsin types [15-17]. In addition, despite BR's excellent potential for generating energy, it has been observed that among those isolates analyzed that harbor the H^+ ^pump, only a few are able to produce large amounts of BR protein in culture [14-16,18,19]. Indeed, in some isolates, the gene encoding BR appears to be present but not expressed [16].

The wide but patchy distribution of rhodopsin genes among haloarchaeal lineages led Ihara *et al. *to propose that the four types of rhodopsin functionality (BR, HR, SRI and SRII) were present in the last common ancestor of all haloarchaea [20], a hypothesis that would suggest rhodopsin genes have been repeatedly lost in different lineages. However, a similar patchy distribution of *type 1 *microbial rhodopsin genes among bacteria and archaea more generally has been interpreted as the result of lateral gene transfer (LGT), sometimes spanning the boundaries between the three domains of life [8,21-23]. We considered it likely that the patchy distribution of rhodopsin gene contents might even more likely reflect episodes of LGT and gene loss, the evidence for which would be incongruent gene phylogenies and unstable gene arrangements within the haloarchaea, the later already evident from comparative genomics but the former not yet shown.

Here we present additional gene arrangement data, and compelling evidence for instances of gene gain by LGT, using phylogenetic analyses of the BR and HR functional types and comparison to the RNA polymerase B' subunit phylogeny (RpoB'). We interpret these results in terms of the physiology of the organisms, and use comparative genomics to infer involvement of certain additional loci in regulation of rhodopsin biogenesis.

## Results

### RpoB' protein phylogeny

**Figure 1:**
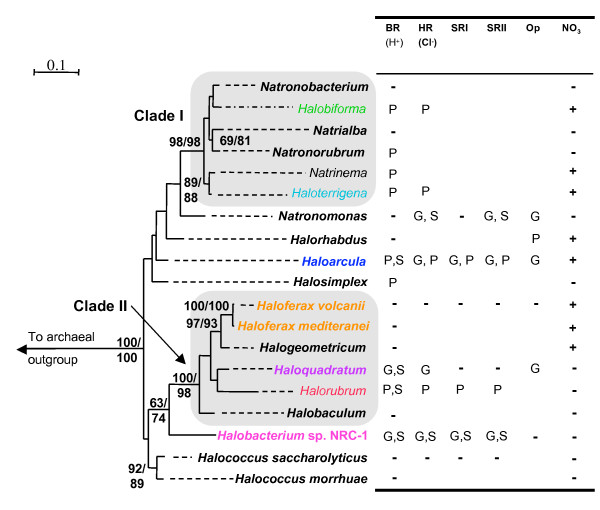
Best maximum likelihood tree (IQPNNI, WAG, estimated shape parameter α with 8 rate categories + invariable sites) of the RNA polymerase B' subunit (435 aa positions) for members of the halophilic archaea. Bootstrap values were obtained using proML and IQPNNI and are shown in that order. Only those nodes that have a bootstrap value greater than 70% are displayed. Branch lengths are extended by dashed lines such that the taxa representing a particular haloarchaeal genus can be readily associated with the corresponding row in the table, which indicates rhodopsin and nitrate reduction presence/absence within that taxonomic division. The code used in the table to denote the presence/absence of bacteriorhodopsin (BR), halorhodopsin (HR), sensory rhodopsins I and II (SRI and SRII), opsin paralog of unknown function (Op) and nitrate reduction (NO_3_) is: G = genome sequence, P = Gene product, S = spectroscopic protein assay, - = Absent. A blank box indicates that information on presence or absence is not available. The groups outlined by shaded boxes represent well-supported clades of haloarchaea. Note that in most cases a single RpoB' sequence is used to represent an entire genus and the representative taxa and accession number can be found in the methods section.

A phylogeny of RNA polymerase B' subunit (RpoB') was constructed as a reference tree (Figure 1). The utility of this marker for haloarchaea was demonstrated previously and a significant collection of RpoB' sequences exists for comparative analysis [24]. The RpoB' phylogeny presented here is similar to that previously inferred in Walsh et al. [24] and reconstructed two strongly supported monophyletic groups, the Clade I and Clade II haloarchaea, within a collection of less well resolved haloarchaeal lineages (Figure 1). Analysis of the RpoB' protein from the recently sequenced genome of *Haloquadratum walsbyii *DSM 16790 [25] places this organism among the Clade II haloarchaea with strong bootstrap support. The position of *Halobacterium *sp. NRC-1 as sister to Clade II haloarchaea was unresolved [24].

### Distribution of rhodopsin genes

The presence/absence of rhodopsins in different haloarchaeal genera determined from this study and others, was mapped to the RpoB' phylogeny in Figure 1. Note that in most cases a single sequence was used to represent an entire genus. The identity of the specific sequence included in the phylogeny is described in the methods. In several genera rhodopsins are not present in all members. For example, a bacteriorhodopsin gene PCR product was not obtained from *Haloterrigena turkmenica*, however other members of this genus have been found to harbor this rhodopsin type, and therefore the presence of this rhodopsin type in *Haloterrigena *is assumed. The presence/absence of the bacteriorhodopsin gene, or the BR protein itself as assayed in other studies [16,17], was known for 13 of the 17 genera represented in the RpoB' tree. We attempted to amplify the *bR *gene from representatives of those genera where the presence of BR was unknown. Partial rhodopsin gene sequences were obtained in this study from *Halorhabdus utahensis*, *Natronorubrum *str. Tenzan 10, and *Halosimplex carlsbadense *(the presence of BR in this organism was noted by Dr. Kunio Ihara, personal communication). Additional sequences were also obtained for additional haloarchaea (supplementary Figure 2). The bacteriorhodopsin gene could not be amplified from *Halogeometricum borinquense, Natronobacterium *str. SSL6, *Natrinema versiforme*, *Haloterrigena turkmenica*, nor various isolates that display close relation to *Halorubrum lacusprofundi *cultivated in a salt spring [26]. Kamekura *et al*. previously documented that some members of the genus *Halorubrum *are unable to produce BR and appear to lack bacteriorhodopsin genes [16]. In some cases the presence/absence of other rhodopsin types was known from genome sequences. For example, *H. walsbyi *is non-motile and lacks sensory rhodopsins but encodes the gene for *hR *[25]. *Natronomonas pharaonis *encodes HR and SR types but lacks a bacteriorhodopsin gene [16,27]. Organisms of the genus *Halococcus *and *Haloferax *reportedly lack rhodopsin pigments entirely [3,17]. The ability to perform nitrate reduction as an alternative energy source was also mapped onto the RpoB' reference tree [17,28]. Nitrate reduction is considered in the discussion as part of a hypothesis for certain cases of variation in rhodopsin gene content and expression.

**Figure 2:**
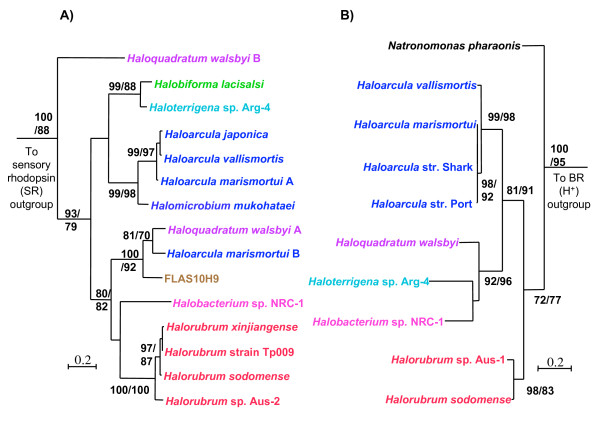
Best maximum likelihood trees (IQPNNI, WAG, estimated shape parameter α with 8 rate categories + invariable sites) of **(A) **the BR protein (197 aa positions) and **(B) **HR protein (249 aa positions) for members of the haloarchaea. Bootstrap values were obtained using PHYML and IQPNNI and are shown on this order in the tree. Only bootstrap values greater than 70% are displayed. Clades that represent a single genus are colour coded. The *H. walsbyi ***B **sequence (purple) in **(A) **represents a rhodopsin of unknown function.

### Bacteriorhodopsin protein phylogeny

The mapping of rhodopsins and nitrate reduction onto the RpoB' phylogeny indicate that these functions are patchily distributed among the haloarchaea and are not confined to any specific clade. Such a pattern of gene presence/absence is most easily interpreted in terms of LGT and gene loss, but only complete genomes sequence data can show that genes are truly absent. Strongly supported phylogenetic incongruence provides independent and often more compelling evidence for LGT. Figure 2a shows a maximum likelihood phylogeny of inferred BR proteins within haloarchaea. (Note that *H. walsbyi *DSM 16790 genome revealed a novel rhodopsin type, indicated in Figure 2a as *H. walsbyi *copy B, that did not cluster within any of the four known functional groups, but branched basal to the proton pumps (as originally indicated in Bolhuis et al. [25])

Comparison of the BR phylogeny to RpoB' demonstrated several similarities. For example, the Clade I haloarchaea represented by *Halobiforma lacisalsi *and *Haloterrigena *sp. Arg-4 were also monophyletic in the BR tree. The position of the BR proteins from *Haloarcula *and *Halomicrobium *are unresolved with respect to the rest of the haloarchaea, but grouped together with strong bootstrap support. The Clade II haloarchaea are represented by *Halorubrum *and *Haloquadratum*, both of which formed a clade with *Halobacterium *sp. NRC-1 with moderate support. This grouping supported the relationship observed between these three genera in the RpoB' tree, which can be corroborated by physiological information regarding phototrophy that will be discussed later. The placement of *H. walsbyi *copy A among other Clade II related haloarchaea in the BR phylogeny was further supported by the addition of incomplete sequences recovered from a environmental BR diversity study of the crystallizer pond in Santa Pola, where *Haloquadratum*-type organisms are the dominant haloarchaea [29]. These *Haloquadratum*-related sequences, in addition to other incomplete BR gene sequences from both the database and this study are presented in additional file 1 because their short lengths decreased the number of amino acid positions available for phylogenetic analyses.

Well-supported discrepancies between the RpoB' and the BR phylogenies were also observed, however. Most notably, the genome of *Haloarcula marismortui *[30] was found to encode two proton-pump-like genes. One copy (*H. marismortui *A) grouped amongst those BR proteins known to be common to the genus *Haloarcula*, while the other (*H. marismortui *B) formed a strongly supported clade with *H. walsbyi*. In addition the BR sequence from an environmental fosmid clone (FLAS10H9) obtained in this study formed a robust grouping within those BR proteins derived from the Clade II haloarchaea. Evolutionary analyses of 5 ORFs spread across the genomic fragment (as opposed to clustered) from FLAS10H9 (additional files 2 and 3) indicate that this genomic fragment did not originate from a Clade II haloarchaeon. In addition, the %GC of FLAS10H9 (~70%) differs substantially from *H. walsbyi *(~48%), the BR protein most closely related to that encoded on FLAS10H9.

### Bacteriorhodopsin sequence diversity

To assess the overall diversity of the BR sequence types among the haloarchaea twenty incomplete BR sequences referred to above were included in a separate phylogeny (additional file 1). Note that the partial sequences obtained in the study by Pašić *et al*. [31] were not included here due to their decreased length in comparison to the partial sequences used here. This phylogeny lacks 73 aa positions that were present in the BR phylogeny in Figure 2a, resulting in a decrease in the resolution of the tree topology. Although the relationships between groups of haloarchaea were less clearly resolved, this analysis provided strong support for the existence of four clades that reflect the evolutionary relationships observed in the RpoB' tree (Figure 1) with the exception of the LGT events discussed later. These clades contain both environmental sequences from a previous study of the saltern in Santa Pola, Spain, in addition to sequences amplified from uncharacterized haloarchaeal strains cultivated from the same location, indicating that the known diversity of BR sequences from this location is well represented by sequences from cultivated haloarchaea. Note that a similar pattern was observed for BR sequences from a solar saltern facility in Slovenia [31], suggesting that BR diversity from thalassic solar salterns is relatively well described.

### Halorhodopsin phylogeny

To determine whether different rhodopsin types displayed the same evolutionary history, we constructed a phylogeny for HR proteins (Figure 2b). The HR protein phylogeny appeared incongruent with both the BR and the RpoB' phylogenies. The most striking incongruence was a grouping of *Haloterrigena *sp. Arg-4, a Clade I haloarchaeon, with *H. walsbyi *and *Halobacterium *sp. NRC-1. Although *Haloterrigena *sp. Arg-4 is not represented in the RpoB' phylogeny, its 16S sequence (AB009624) was 94% identical to the Clade I haloarchaeon *Natrinema pallidum*, whose RpoB' sequence is present as the representitve for *Natrinema *in Figure 1. The relationship between the Clade II haloarchaea and *Halobacterium *sp. NRC-1 observed in both the RpoB' and the BR protein phylogenies (Figs. 1 and 2a) is disrupted in Figure 2b, where *Haloarcula *HR proteins form a clade with the HR sequences from *H. walsbyi *and *Halobacterium *sp. NRC-1 to the exclusion of *Halorubrum *encoded HR. These observations indicate an alternative evolutionary history for HR, different from that of BR and RpoB'.

### Bacteriorhodopsin-linked regions of haloarchaeal genomes

**Figure 3:**
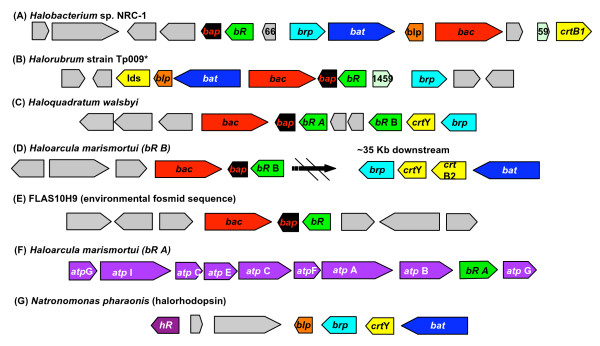
The *bR*-linked regions of five haloarchaeal genomes (plus the *hR *region from *N. pharaonis*. The boxed arrows represent genes that are colour coded according to the following: *bacteriorhodopsin *(*bR*, green), *bR *gene activator (*bat*, blue), *bR *related protein (*brp*, teal), *bR *linked protein (*brp*, orange), *bR *associated chaperone (*bac*, red), *bR *associated protein, retinal synthesis genes (yellow), *atp *synthetase (*atp*, purple), cellular functions unrelated to BR function (grey) and their functionality is indicated in the text. The direction of transcription is indicated by the orientation of the arrow. The structure of the bacteriorhodopsin gene cluster is variable between haloarchaeal genera, but shares pronounced similarities between those lineages that group together in the RpoB' phylogeny.

In the genome of *Halobacterium *sp. NRC-1 [32] the gene encoding *bR *is adjacent to genes important to its function and expression (Figure 3), all of which are transcriptionally co-regulated under micro-oxic conditions when accompanied by exposure to light [33]. Under these conditions the bacterio-opsin activator protein (Bat) binds to the upstream activator sequences of genes belonging to the *bR *regulon, which include the *bat *gene itself, bacterio-opsin related protein (*brp*) and phytoene synthase (*crtB1*). The last two are responsible for catalyzing steps in the biosynthesis of the retinal chromophore required for rhodopsin function. The Bat protein also induces transcription of the gene encoding the bacterio-opsin linked protein (*blp*) whose function is unknown [34].

**Figure 4:**
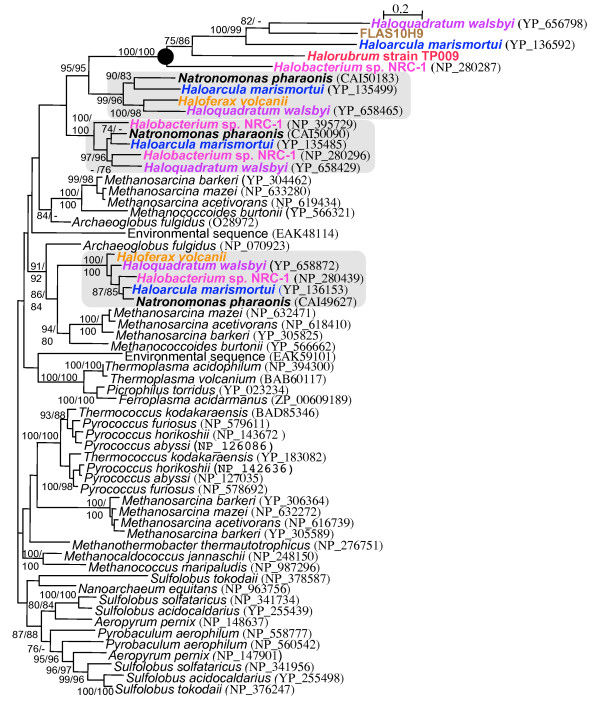
Phylogeny of the archaeal CDC48 protein family (IQPNNI, WAG + estimated shape parameter α with eight rate categories plus invariable sites for 491 aa positions). The clade representing the Bac proteins associated with the *bR *gene are indicated by a black circle. Other groups of haloarchaeal paralogs are indicated by shaded boxes and bold taxa names. Bootstrap values were obtained using phyML and IQPNNI and are displayed in that order. Only bootstrap values greater than 70% are displayed.

Bacteriorhodopsin-linked genomic regions are also available for two other sequenced haloarchaeal genomes (Figure 4), *H*. *marismortui *[30] (with two *bR *genes) and *H. walsbyi *DSM 16790 [25]. Although the sequenced genome of the haloarchaeaon *Natronomonas pharaonis *[27] does not harbour a *bR *gene, it encodes a Cl^- ^pumping rhodopsin (halorhodopsin, HR). The genomic context of *hR *in *N. pharaonis *suggests that both HR and BR have converged on similar solutions in that the genes encoding these rhodopsins are linked to others important to their biogenesis (Figure 3). Note that there were no linkage patterns displayed among *hR *genes from the remaining haloarchaeal genomes.

In addition we have sequenced *bR *gene regions from two fosmid clones, one from *Halorubrum *strain TP009 (Clade II haloarchaeaon) isolated from a solar saltern in Santa Pola, Spain and the other, FLAS10H9, from metagenomic library created from the same solar saltern sampled at the same time. A cultured organism from the genus *Halorubrum *was chosen because of its relationship to the Clade II haloarchaea, and in addition *Halorubrum *represents an important haloarchaeal division for which no sequenced genome is available [26,31,35]. Clone FLAS10H9 was chosen because it encodes a *bR *gene highly similar to that of *H. walsbyi*, although the surrounding genomic context of this clone is different evolutionary relationships (additional files 2 and 3).

In these haloarchaeal genomes, the *bR *gene was observed in three general linkage states (Figure 3). The first state is that exhibited by *Halobacterium *sp. NRC-1, *H. walsbyi *DSM 16790, and *Halorubrum *str. TP009, in which *bR *was linked to genes required for its biogenesis, although there were slight differences in the genomic structure and content. Rearrangement of these genes is observed in all three organisms, and in some cases genes were substituted in or out. For example, *blp *and *bat *are not linked to *bR *in the gene cluster in *H. walsbyi*, although a second proton-pump-like gene was present. Additionally, each organism encodes genes for different steps along the carotenoid synthesis pathway in their *bR *gene cluster (genes indicated in yellow, Figure 3).

These three genomes had three genes in common that were linked to *bR*. The *brp *gene encodes the enzyme that catalyses the final step in retinal synthesis [34]. Two others are open reading frames (ORFs) that were also implicated in BR biogenesis in previous studies limited to *Halobacterium *sp. NRC-1 [36,37]. These ORFs encode a paralog of Cdc48, a AAA-ATPase cell division cycle protein that we have chosen to designate bacterio-opsin associated chaperone (*bac*, see next section) and a small open reading frame (50–70aa) hereafter designated as bacterio-opsin associated protein (*bap*). Although the presence of these genes have been described in isolated instances, our designation is based on common occurrence and likely conserved function. Further analysis of the inferred Bap protein with transmembrane prediction [38,39] and amino acid backbone modeling (using 3D position specific scoring matrix fold recognition server, [40]) indicated a two pass transmembrane protein structure.

The second general linkage pattern was simply the *bR *gene clustered with *bap *and *bac *(Figure 3). This pattern was observed for *H. marismortui *(copy B) and also the *bR *gene from the environmental fosmid clone FLAS10H9. The third pattern linked *bR *and genes encoding for the subunits of the H^+^-ATPase. This was observed only for the copy of bR in *H. marismortui *(copy B) that appears ancestral to the genus *Haloarcula *(Figure 2). A similar pattern has been described for genomic fragment from *Haloarcula japonica*, in which a gene encoding an H^+^-ATPase subunit lies upsteam of a *bR *gene [18].

### Cell division cycle 48 protein phylogeny

Phylogenetic analysis was also performed on the *cdc*48 paralogs frequently linked to the *bR *gene, which we designate *bac *(bacterio-opsin associated chaperone) (Figure 4), in the context of a broad sampling of archaeal *cdc*48 genes. Cdc48 comprise a family of proteins described as ATPases associated with a variety of cellular activites (AAA-ATPases) [41]. Homologs of the Cdc48 protein family are present in all three domains of life, and as their name suggests are associated with many different functions, such as membrane fusion, proteolysis, chaperone-like activites and cell division. The genes encoding these proteins are widespread in archaeal genomes, where they are usually present in multiple copies (Figure 4).

A phylogeny of selected archaeal Cdc48 homologs, including all *bR*-linked *bac *genes, is represented in Figure 4. Sequences tend to fall into well-supported groups but weak backbone support for the overall phylogeny leaves relationships between these groups unresolved. For instance, the separation into four groups of Cdc48 paralogs originating from the Halobacteriales are resolved with regards to each other and well supported to the exclusion of other euryarchaeotes, but their position within the euryarchaeotes is uncertain. The protein sequences of the Bac paralog of Cdc48 – those that are linked to the gene encoding BR in haloarchaeal genomes (Figure 4) – fall into one of these well-supported clusters and are represented by the 5 sequences in the clade marked by the black dot, which are associated with long branches. Three Cdc48 paralogs not linked to *bR *are found in the each of the sequenced genomes, including *Haloferax volcanii *and *Natronomonas pharonis*, both of which lack *bR *and the *bac *paralog of Cdc48. It is these additional genes which make up the remaining three well-supported clusters within the Cdc48 phylogeny. Phylogenies within these clusters are generally congruent to each other and well supported at most nodes, but disagree with the well-supported Bac protein phylogeny.

The topology for the Bac protein is incongruent with the RpoB' tree. For example the grouping between *Haloarcula marismortui*, FLAS10H9 and *Haloquadratum walsbyi *supports a similar discrepancy in the BR phylogeny (Figure 2a). However, the phylogeny for the Bac paralog must be considered with some caution due to long branch attraction (LBA) artifacts [42]. An additional phylogenetic analysis of a Cdc48 alignment constructed using only the more conservative regions of the protein yielded similar support and topology for the Bac encoding paralogs providing further support for the relationship shown in Figure 3 and its incongruence to phylogenies for those Cdc48 paralogs not associated with BR (data not shown).

## Discussion

In this study we performed comparative genomic and phylogenetic analyses to address questions regarding the evolution of BR-based phototrophy and the erratic distribution of rhodopsins among haloarchaea. The distribution of rhodopsins mapped onto the RpoB' phylogeny (Figure 1) of the haloarchaea supported previous notions that these proteins are patchily distributed rather than present in a specific clade of haloarchaea and absent in others. Here we attempt to explain potential factors responsible for the patchy distribution of rhodopsins within this lineage of archaea and place physiological information of these organisms into an evolutionary context with regards to phototrophy.

### Bacteriorhodopsin-based phototrophy in haloarchaea

The broad distribution of the different functional types of rhodopsins, including BR, among haloarchaea has lead to suggestions that these proteins were present in the ancestor of this archaeal group [20]. The phylogeny in Figure 2a and its similarities to the RpoB tree (Figure 1) provides evidence in favor of this hypothesis for the proton pumping BR types. Although BR may have been an ancestral feature of haloarchaea, our comparative and phylogenetic analyses revealed a correlation between those isolates capable of producing high concentrations of BR in the membrane and evolutionary relatedness. The phylogeny inferred from RpoB' (Figure 1) indicated that *Halobacterium *sp. NRC-1 forms a group with the Clade II haloarchaea, which contains both *Halorubrum *and *Haloquadratum*. Previous studies have documented that isolates from these genera highly express BR, more so than those from other haloarchaeal lineages [3,15,16,25,42,43]. Additional evidence supporting this correlation is seen in the structure and composition of the bacteriorhodopsin gene cluster in the genomes of *Halobacterium *sp. NRC-1, *Halorubrum *str. TP009 and *Haloquadratum walsbyi*, where bacteriorhodopsin is linked to genes important for the synthesis of the chromophore retinal and the biogenesis of the BR protein (Figure 3). Furthermore, in the BR protein phylogeny (Figure 2a), the sequence from *Halobacterium *sp. NRC-1 groups together with *Halorubrum *species and *Haloquadratum walsbyi *in a well-supported clade. While this might suggest that the ability to efficiently incorporate BR into energy production evolved in the lineage that gave rise to *Halobacterium sp*. NRC-1 and the Clade II haloarchaea, this hypothesis should be considered with some caution due to the small sampling of cultivars analyzed and the unknown influence of cultivation on their ability to grow phototrophically.

Interestingly, members of *Halorubrum *and *Haloquadratum *were found to represent a significant fraction of solar saltern microbial communities in Slovenian [31] and Spanish [44] crystallizer ponds respectively. The shallow crystallizer ponds represent the final stages of salt production where precipitation occurs, thus resulting in the lowest concentration of oxygen of all the ponds in the solar saltern environment [45]. The dominance of these groups under such conditions hints that rhodopsin-based energy-generating photosystems may play a significant role in the success of these organisms in such ecosystems. In addition, the most dominant bacterium in the Spanish crystallizer pond, *Salinibacter ruber *was shown to produce a novel type of rhodopsin (xanthorhodopsin) that uses carotenoid antennae to absorb across a greater range of wavelengths of light [46].

The observation of refined rhodopsin-based energy-generating photosystems [5,17,25] in some groups is in contrast to those lineages that lack BR and rhodopsin functionality altogether. For example, Kamekura *et al*. documented that some *Halorubrum *species lack BR, suggesting patchy distribution even at the genus level [16]. If it is assumed that BR was present prior to the divergence of Clade II haloarchaea, then this patchy distribution is best explained by multiple independent losses. Mukohata *et al*. [47] previously suggested loss of rhodopsins in *Haloferax*, a conclusion which is also supported by our analyses here. Rhodopsin pigments have not been detected in any of the members from the genus *Haloferax *[16,47], or the genome of *Haloferax volcanii*, currently being sequenced by TIGR [48]. *Haloferax *is also a terminal member of the Clade II haloarchaea (Figure 1) suggesting that rhodopsins were lost recently, prior to the divergence of this genus.

Closer examination of the genus *Haloferax *reveals that many of its members have an alternative mechanism to generate energy that phototrophic members of Clade II appear to lack [17,49]. The ability of certain *Haloferax *members to perform nitrate reduction (Figure 1) suggests they are able to cope with long periods of anoxic conditions, which is interesting in light of the inability of haloarchaea to produce retinal in the complete absence of oxygen [5]. One potential hypothesis for the loss of rhodopsins in *Haloferax *may be that the ancestor of this group adapted to prolonged anoxic conditions, during which rhodopsin-based energy would be frequently unavailable.

### LGT and loss: the patchy distribution of rhodopsins

**Figure 5:**
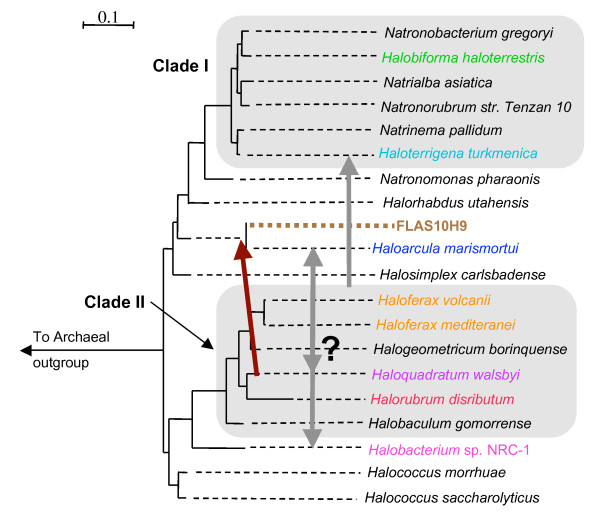
Lateral gene transfer events of bacteriorhodopsin, bacterio-opsin associated chaperone and bacterio-opsin associated protein (dark red arrow) and halorhodopsin (gray arrows) inferred from this study (see results section) mapped onto a representation of the RpoB' reference tree from Figure 1. Branch lengths are extended by dashed lines, and the environmental fosmid FLAS10H9 (brown) is given an arbitrary position on the tree based on phylogenetic analyses on selected ORFs encoded on the genomic fragment. Those phylogenies (additional files 2 and 3) indicate that FLAS10H9 does not originate from a Clade II haloarchaeon, while some ORFs group with the genus Haloarcula. The bacteriorhodopsin and *bac *genes of FLAS10H9 (Figure 2a and 4) share affiliation with the Clade II haloarchaea, particularly *Haloquadratum walsbyi*. Given the limited amount of sequence data for the HR types, it is difficult to resolve the donor and recipient groups for transfer events that gave rise to the topology observed in Figure 2b

The probable loss of rhodopsins in *Haloferax *and *Halorubrum *highlights a general question regarding the evolution of this group of proteins in haloarchaea and their patchy distribution. Previously, Ihara et al. [20] suggested that the different types of rhodopsins were present in the last common ancestor of the haloarchaea, which would suggest that the patchy distribution of rhodopsins observed among these organisms is the result of multiple losses. More recently however, several studies have revealed the genetic mobility of genes encoding type 1 rhodopsins among microbes. The work described here provides at least three highly probable cases of LGT within haloarchaea. These events are sketched in Figure 5 and briefly discussed below. Note that the sensory rhodopsins are less well represented in the database and therefore were not included in this study.

### *bR *gene transfer

The comparison of the BR protein phylogeny (Figure 2a) to the RpoB' reference tree revealed two instances where the phylogenies were incongruent. In both *Haloarcula marismortui *and the environmental fosmid FLAS10H9 the bacteriorhodopsin gene was observed in a similar linkage state, where it was found adjacent to the genes *bac *and *bap *(Figure 4). Phylogenetic analyses of the BR and Bac sequences observed in this linkage state revealed that the genes encoding these proteins were likely laterally transferred into both genomes as a unit. It is possible that these LGT events may have occurred independently of each other. However, the unresolved positions of both FLAS10H9 and the genus *Haloarcula *in combination with the grouping of some FLAS10H9 ORFs with *Haloarcula *(additional file 2) suggests the most parsimonious interpretation as a single transfer into the ancestor of these organisms from a *Haloquadratum*-releated lineage (Figure 5).

The co-transfer of genes encoding rhodopsins with their linked complements was also observed in a recent study by McCarren and DeLong [23]. In that study, genes encoding components of the retinal biosynthesis pathway were shown to co-migrate with proteorhodopsin genes between divergent microbial lineages to deliver a fully functional photosystem to the recipient organism. While the genes co-transferred with bacteriorhodopsin here do not seem to play a direct role in retinal synthesis, perhaps because the recipient haloarchaeal genome already encoded such capabilities, the ability of Bac and Bap to confer a rhodopsin specific selective advantage is discussed below.

### Incongruence of tree topologies for BR and HR

Multiple scenarios infer a minimum of two LGT events involving the Cl^- ^pumping types to account for incongruent phylogenies between this protein and BR (figs. 2 and 5). The number and direction of *hR *transfers is difficult to interpret due to the unresolved position of *Haloarcula *in the RpoB' phylogeny (Figure 1) and the limited amount of sequence data for the Cl^- ^pumping types.

The phylogenetic evidence presented here strongly support our hypothesis that lateral gene transfer in conjunction with gene loss has contributed to the patchy distribution of rhodopsins among haloarchaea. While particular rhodopsin functional types (namely HR) may have first arisen in different haloarchaeal lineages and then spread amongst other members of this group by LGT, we cannot rule out more complex scenarios in which all rhodopsins were present in the haloarchaeal cenancestor but also have a more recent history of between-lineage LGT. The acquisition of additional sequence data for the BR and HR proteins in addition to the sensory rhodopsin types will provide a more accurate picture of this possibly quite complex history. The haloarchaeal origin of certain rhodopsin paralogs (halorhodopsin, sensory rhodopsin) in the genome of the hyperhalophilic bacterium *S. ruber *may represent an analogous example of the spread of rhodopsin-functionality by LGT [21].

### Frequent linkage suggests Bac and Bap play a role in BR function

The observation of the frequent linkage of gene sets over broad evolutionary distances suggests shared function. The genes we describe as *bac *and *bap *were linked to the bacteriorhodopsin gene at a higher frequency (Figure 3) than those genes previously shown to be important to the function and expression of the proton pump, suggesting an important role for these proteins in BR-based phototrophy. The Bac protein displayed homology to the Cdc48 protein family, a multifunctional group of proteins that belong to the AAA-ATPases [41]. The significance of Bac to BR function is supported by multiple lines of evidence; frequent linkage, co-transfer with the *bR *gene (Figure 4) and the striking absence of Bac paralogs in those haloarchaeal genomes that lack BR. In relation to other Cdc48 proteins in the domain archaea, the Bac paralogs are associated with unusually long branch lengths, suggesting that their function is being co-opted to a cellular role that is uniquely haloarchaeal (e.g. BR). In addition to this evidence, a recent bioinformatics analysis of the *Halobacterium*. sp. NRC-1 genome by Reiss *et al*. [36] found homology for the transcriptional activation sequence for the BR regulon upstream of the *bac *gene. The function of Cdc48 proteins in archaea is relatively unexplored. Golbik *et al*. [50,51] implicated a Cdc48 paralog of *Thermoplasma acidophilum *in chaperone-like activities. How BR transverses the lipid bilayer has yet to be completely elucidated [52], and we suggest that Bac plays a chaperone-like role in BR protein translocation and membrane insertion.

The *bap *gene is also frequently linked to *bR *and encodes a haloarchaeal-specific conserved protein that has only been detected in a *bR *related genomic context. Using integrated microarray and proteomic data, Baliga *et al*. [37] found a decrease in Bap protein expression when the transcriptional regulator of the BR regulon (Bat) was knocked out. While no functional role could be assigned to this protein on the basis of sequence similarity, Bap shared structural similarity with the two-pass transmembrane component of sensory rhodopsin transducers. Transducer proteins (Htr) have two main functions with regards to sensory rhodopsins (SR). These proteins help to relay the phototactic signal received from SR to the flagella and also help to insert and align SR in the membrane in a higher order structure (2:2 ratio of SR to Htr) [53]. In the absence of Htr, SR is inserted into the membrane with far less efficiency, and membrane insertion activity can be restored by the addition of the DNA sequence encoding the signal sequence peptide of BR [54]. Perhaps Bap plays a similar role to that of Htr in membrane insertion, although no two-pass transmembrane protein has been observed in the structure of the purple membrane, indicating that such a role would be transient.

## Conclusion

Bacteriorhodopsin and its several homologs have long been considered defining features of the haloarchaea, and have been developed into superb model systems by structural biologists and photophysiologists. But they are not limited in their occurrence to haloarchaea, nor are they universally present among the latter. Furthermore, these functionally differentiated proteins play distinct physiological roles and appear to be regulated differently within and between the haloarchaeal lineages in which they are found.

In a recent review of photosynthesis Bryant and Frigaard commented that the reaction centers employed by photosynthetic bacteria are a more efficient means of obtaining energy than rhodopsin-based phototrophy, due to both the efficiency of converting photon energy to ATP and the presence of large amounts of chromophore in a single unit of reaction center [55]. Consequently, BR-based phototrophy in haloarchaea likely acts as a supplementary energy source, which some lineages may exploit better than others. Presumably these differences in gene content, organization and expression reflect such exploitation, are adaptive and clearly, as we show here, they can arise through lateral gene transfer.

Several recent studies suggest that rhodopsin genes should be considered part of the "habitat genome", a pool of genes useful for adaptation to a particular set of environmental constraints [8,21,23,56]. Clearly some haloarchaeal lineages have assembled refined rhodopsin-based energy systems in response to such constraints, while others have adapted to different ecological niches where rhodopsin functionality may or may not be part of such a habitat genome. Consequently, it should not be surprising that rhodopsin functionality is frequently lost, gained by LGT, or even that the broad distribution of certain functional-types may be the result of rhodopsin gene mobility.

## Methods

### Phylogenetic analyses

RpoB', BR, HR and Cdc48 sequence data was obtained from the protein database and blast searches at NCBI [57]. The accession numbers for BR and Cdc48 sequences are listed on additional file 1 and Figure 4.

RpoB' accession numbers: *Halococcus saccharolyticus *(CAH10896), *Halococcus morrhuae *(CAH10913), *Halobacterium *sp. NRC-1 (NP_281213), *Halobaculum gomorrense *(CAH10894), *Halorubrum disributum*(CAH10902), *Haloquadratum walsbyi *(YP_659080), *Halogeometricum borinquense *(CAH10899), *Haloferax mediteranei *(CAH10898), *Haloferax volcanii *[48], *Halosimplex carlsbadense *(CAH10903), *Haloarcula marismortui *(YP_136946), *Halorhabdus utahensis *(CAH10900), *Natronomonas pharaonis *(CAH10910), *Haloterrigena turkmenica *(CAH10905), *Natrinema pallidum *(CAH10907), *Natronorubrum str. Tenzan 10 *(CAH10911), *Natrialba asiatica *(CAH10906), *Halobiforma haloterrestris *(CAH10895), *Natronobacterium gregoryi *(CAH10908).

HR accession numbers: *Natronomonas pharaonis *(P15647), *Haloarcula vallismortis *(P94853), *Haloarcula marismortui *(AAV46572), *Haloarcula *str. Port (Q48315), *Haloarcula *str. Shark (Q48314), *Haloquadratum walsbyi *(CAJ53165), *Haloterrigena *sp. Arg-4 (O93741), *Halobacterium *sp. NRC-1 (NP_279315), *Halorubrum *sp. Aus-1 (CAA49773), *Halorubrum sodomense *(O93742).

*Halorhabdus utahensis *opsin paralog (EF558560).

Haloarchaeal fosmid clone accession numbers: Uncultured haloarchaeon FLAS10H9 (EF558547, EF558548). *Halorubrum *strain TP009 (EF558549, EF558550, EF558551, EF558552).

Automated alignment for each protein was performed using ClustalX [58], after which, alignment was manually adjusted with MacClade 4.06 [59]. Rhodopsin alignments were performed using a previous alignment as reference [20] to remove hyper-variable loops and other unaligned regions. The accession numbers of the sensory rhodopsin sequences used to root the BR phylogeny were YP_134805, NP_280508, BAA75203, YP_137680. The accession numbers of the BR sequences used to root the HR phylogeny were AAV47867, CAJ51147, AAS15567. Phylogenetic analyses were performed using IQPNNI [60]. Models of evolution are indicated in the Figure legends. The protein substitution matrix WAG was used for all phylogenies [61]. The confidence of each node was determined by assembling a consensus tree of 100 bootstrap replicates using both IQPNNI and PHYML [62,63].

### BR gene PCR

The sequences of the primers used to amplify the BR gene from Papke et al. [29] are as follows: 381–401 forward (5'-GAC TGG TTG TTY ACV ACG CC -3') and 795–814 reverse (5'-AAG CCG AAG CCG AYC TTB GC-3'). PCR amplification reactions were carried out in a final volume of 50ul containing 1–5 ng of template DNA, 1 × PCR buffer, 1 ul of 10 mM deoxynucleoside triphosphate, 2 ul of 50 mM MgCl_2_, 2 ul of a solution of each primer (F/R) at a concentration of 10 uM and 0.5 ul of *Taq *polymerase (Invitrogen).

BR gene fragments were amplified with an initial denaturation at 95 C for 3 minutes, 30 cycles with a denaturation at 95 C for 45 seconds, primer annealing at 54.8 C for 45 seconds, and primer extension at 68 C for 30 seconds. Thermal cycling was followed by a final terminal extension step at 68 C for 5 minutes. Multiplex PCR of fosmid clone preps to identify BR-containing clones was carried out using the same approach. In most cases PCR products were directly sequenced, except for *Halorhabdus utahensis *where the PCR product was cloned before sequencing (TOPO-TA; Invitrogen). Sequencing was performed using ABI Big Dye-terminator chemistry on an ABI 377 instrument. The sequences were trimmed and analyzed using Sequencher 4.2.2.

### Cultivation methods

*Halorubrum *str. TP009 and other strains used in this study were isolated from a solar saltern in Santa Pola, Spain. For cultivation methods see Papke et al. [64].

### Fosmid library construction and sequencing

Genomic DNA was obtained from *Halorubrum *str. TP009 using the protocol of Wilson [65]. The fosmid FLAS10H9 was obtained from the environmental fosmid library of the Santa Pola saltern described in Legault et al. [56]. Fosmid libraries were constructed using the CopyControl fosmid library production kit from Epicentre (Madison, WI) following the protocol of the manufacturer. Fosmid clones were miniprepped using either alkaline lysis prep on grown cultures with the R.E.A.L. Prep 96 plasmid kit (QIAGEN, Chatsworth, CA). Clones containing BR genes were identified by PCR screening as described above. Subcloning of fosmids was done using the TOPO Shotgun subcloning kit (Invitrogen). Subclones were sequenced using DYEnamic™ ET Dye Terminator Kit (MegaBACE) and a MegaBACE™ 1000 (Amersham). The level of sequencing coverage for each fosmid was: 13.3X for TP009 and 8.2X for Flas10h9. Low-quality regions and gaps were fixed by PCR. The fosmids were assembled using phredPhrap, and open reading frames (ORFs) were identified using the fgenesB option under operon and gene finding in bacteria at [66] with *Halobacterium *sp. NRC-1 used as the model genome for the search. ORFs were annotated using blastx.

## Authors' contributions

AKS was responsible for the majority of the molecular methods, sequence analyses and drafting of the manuscript. Note that the environmental fosmid clone FLAS10H9 was obtained from the study in [56]. DAW was responsible for the phylogenetic analysis of RpoB' and critical revision of the manuscript. EB helped with the Cdc48a alignment, interpretation of phylogenies and manuscript revision. FR-V contributed to data acquisition and interpretation. WFD helped to draft the manuscript. RTP conceived the study, participated in its coordination and critical revision of the manuscript.

## Supplementary Material

Additional file 1 Phylogeny of partial BR sequences from the database in addition to sequences obtained in this study Click here for file

Additional file 2 Phylogenies of selected ORFs from the environmental fosmid FLAS10H9 Click here for file

Additional file 3 Concatenated phylogeny of selected ORFs from the environmental fosmid FLAS10H9 Click here for file
