# Supplementary material for: Evolution of rhodopsin ion pumps in haloarchaea
Source: BMC Evol Biol. 2007 May 18;7:79. doi: 10.1186/1471-2148-7-79 (PMC1885257; doi:10.1186/1471-2148-7-79)
Supplement: Additional file 1 — Phylogeny of partial BR sequences from the database in addition to sequences obtained in this study [file 1471-2148-7-79-S1.pdf]

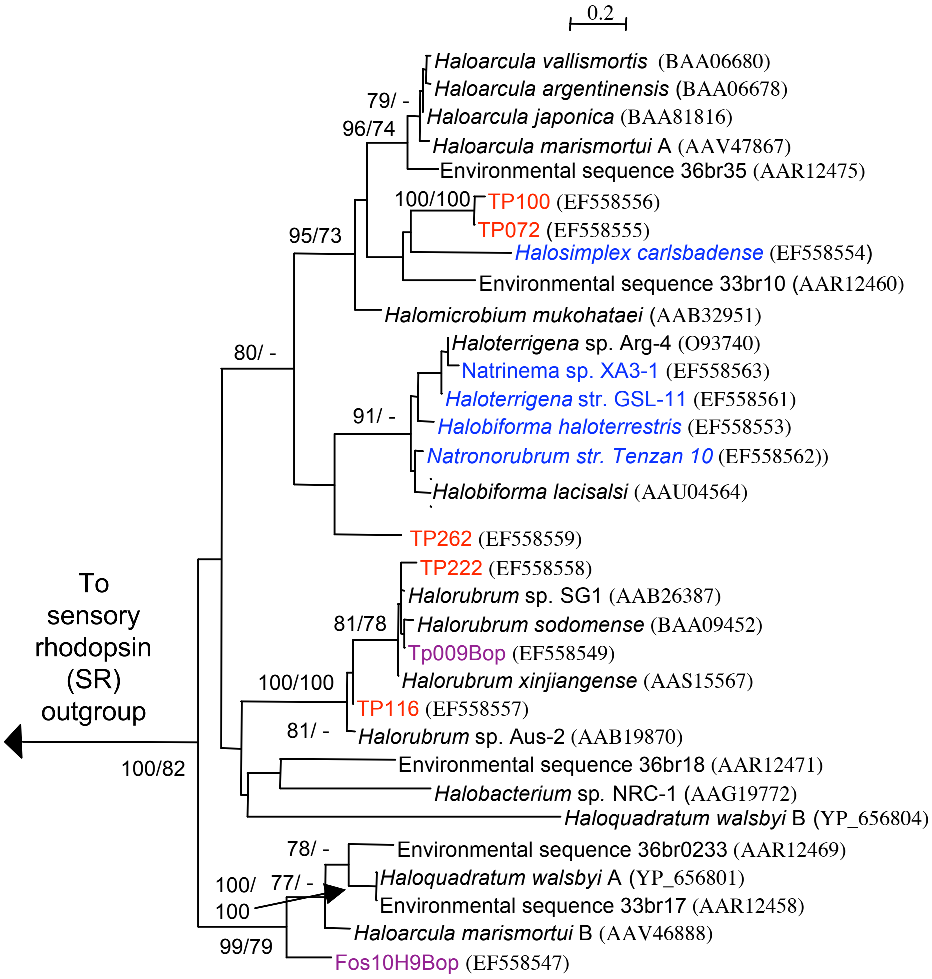

Additional file 1. Best maximum likelihood tree (IQPNNI, WAG, estimated shape parameter a with 8 rate categories + invariable sites) of the BR protein (124 aa positions) including partial sequences for members of the haloarchaea**.** Bootstrap values were obtained using PHYML and IQPNNI and are shown on this order in the tree. Only bootstrap values greater than 70% are displayed. Sequences obtained in this study are indicated in colour and include undescribed strains from our collection, cultivated from a solar saltern in spain (red), described haloarchaea (blue) and two BR sequences obtained from fosmid clones. The affect of using fewer amino acid positions is observed by the branching of copy B of *Haloquadratum walsbyi* within the proton pumps, rather than basal as was observed in fig 2a with good bootstrap support (copy B branches with *Halobacterium* sp. NRC-1, disrupting the monophyly of the BR-types.)
