# Supplementary material for: Evolution of rhodopsin ion pumps in haloarchaea
Source: BMC Evol Biol. 2007 May 18;7:79. doi: 10.1186/1471-2148-7-79 (PMC1885257; doi:10.1186/1471-2148-7-79)
Supplement: Additional file 2 — Phylogenies of selected ORFs from the environmental fosmid FLAS10H9 [file 1471-2148-7-79-S2.pdf]

Additional file 2

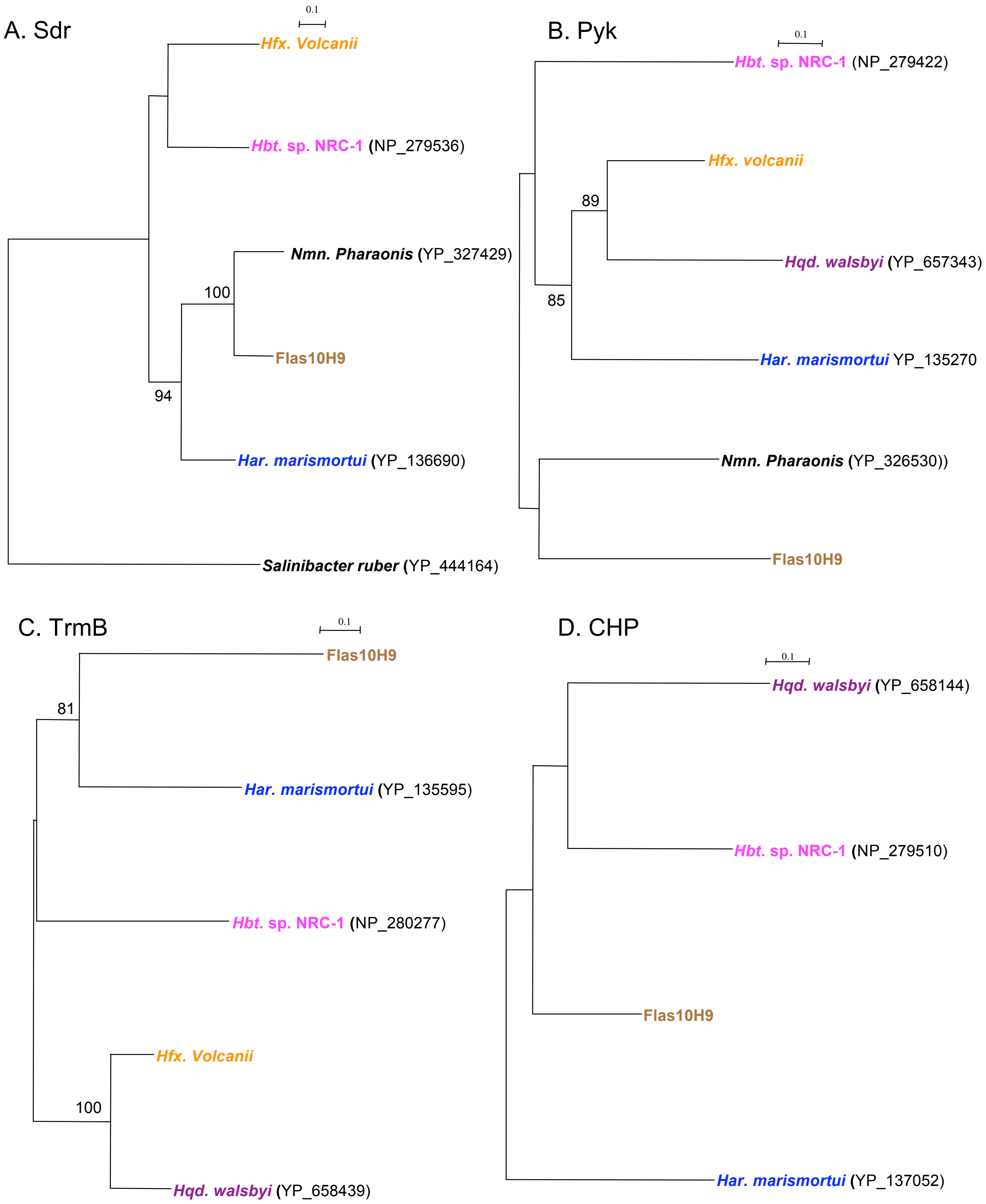

**Additional file 2.** Phylogeny of four ORFs from environmental fosmid FLAS10H9 (IQPNNI, WAG, estimated $\alpha$ parameter with eight rate categories plus invariable sites, bootstrap values also calculated with IQPNNI). A) Short-chain dehydrogenase/reductase (Sdr) superfamily protein (302 aa sites). B) Pyruvate kinase (Pyk) (587 aa sites) C) Sugar-specific transcriptional regulator (TrmB) (347 aa sites) D) Conserved hypothetical protein (397 aa sites)
