# Supplementary material for: Evolution of rhodopsin ion pumps in haloarchaea
Source: BMC Evol Biol. 2007 May 18;7:79. doi: 10.1186/1471-2148-7-79 (PMC1885257; doi:10.1186/1471-2148-7-79)
Supplement: Additional file 3 — Concatenated phylogeny of selected ORFs from the environmental fosmid FLAS10H9 [file 1471-2148-7-79-S3.pdf]

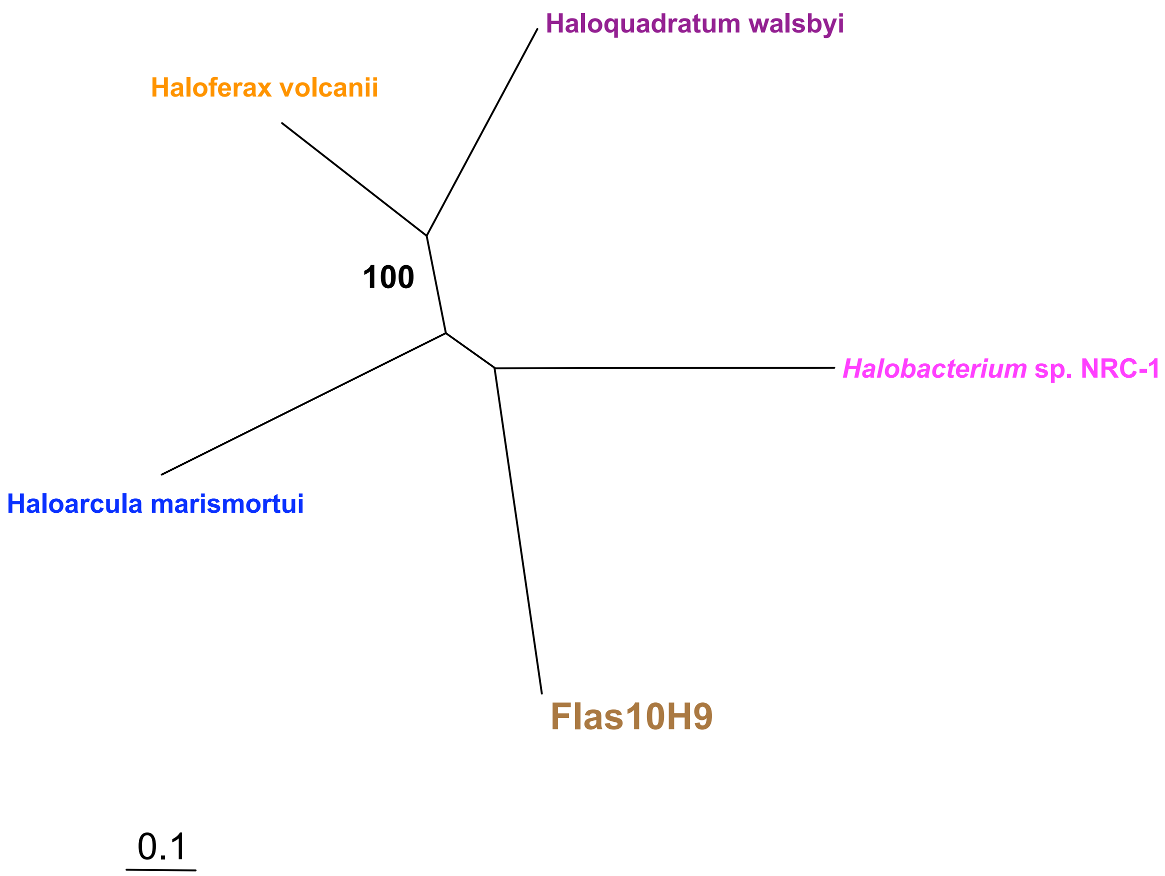

Additional file 3. Concatenated phylogeny of four ORFs (1524 aa) from environmental fosmid FLAS10H9 (IQPNNI, WAG, estimated a parameter with eight rate categories plus invariable sites, bootstrap values also calculated with IQPNNI). The four ORFs concatenated here are Pyruvate kinase (Pyk), Sugar-specific transcriptional regulator (TrmB), Conserved hypothetical protein (CHP) and orotate phosphoribosyltransferase (pyrE). Accession numbers for pyrE are YP_657432 (Haloquadratum walsbyi), CAD37329 (Haloferax volcanii),(YP_135607) Haloarcula marismortui, NP_279512 (*Halobacterium* sp. NRC-1).
